# Alprazolam modulates persistence energy during emotion processing in first-degree relatives of individuals with schizophrenia: a network control study

**DOI:** 10.1038/s41380-023-02121-z

**Published:** 2023-06-23

**Authors:** Arun S. Mahadevan, Eli J. Cornblath, David M. Lydon-Staley, Dale Zhou, Linden Parkes, Bart Larsen, Azeez Adebimpe, Ari E. Kahn, Ruben C. Gur, Raquel E. Gur, Theodore D. Satterthwaite, Daniel H. Wolf, Dani S. Bassett

**Affiliations:** 1 Department of Bioengineering, School of Engineering & Applied Science, University of Pennsylvania, Philadelphia, PA 19104 USA; 2 Department of Neuroscience, Perelman School of Medicine, University of Pennsylvania, Pennsylvania, PA 19104 USA; 3 Annenberg School for Communication, University of Pennsylvania, Philadelphia, PA 19104 USA; 4 Department of Psychiatry, Perelman School of Medicine, University of Pennsylvania, Philadelphia, PA 19104 USA; 5 Department of Neurology, Perelman School of Medicine, University of Pennsylvania, Philadelphia, PA 19104 USA; 6 Department of Radiology, Perelman School of Medicine, University of Pennsylvania, Pennsylvania, PA 19104 USA; 7 Department of Electrical & Systems Engineering, School of Engineering & Applied Science, University of Pennsylvania, Philadelphia, PA 19104 USA; 8 Department of Physics & Astronomy, College of Arts & Sciences, University of Pennsylvania, Philadelphia, PA 19104 USA; 9 Santa Fe Institute, 1399 Hyde Park Rd, Santa Fe, NM 87501 USA; 10 Leonard Davis Institute of Health Economics, University of Pennsylvania, Philadelphia, PA 19104 USA

**Keywords:** Neuroscience, Schizophrenia

## Abstract

Schizophrenia is marked by deficits in facial affect processing associated with abnormalities in GABAergic circuitry, deficits also found in first-degree relatives. Facial affect processing involves a distributed network of brain regions including limbic regions like amygdala and visual processing areas like fusiform cortex. Pharmacological modulation of GABAergic circuitry using benzodiazepines like alprazolam can be useful for studying this facial affect processing network and associated GABAergic abnormalities in schizophrenia. Here, we use pharmacological modulation and computational modeling to study the contribution of GABAergic abnormalities toward emotion processing deficits in schizophrenia. Specifically, we apply principles from network control theory to model persistence energy – the control energy required to maintain brain activation states – during emotion identification and recall tasks, with and without administration of alprazolam, in a sample of first-degree relatives and healthy controls. Here, persistence energy quantifies the magnitude of theoretical external inputs during the task. We find that alprazolam increases persistence energy in relatives but not in controls during threatening face processing, suggesting a compensatory mechanism given the relative absence of behavioral abnormalities in this sample of unaffected relatives. Further, we demonstrate that regions in the fusiform and occipital cortices are important for facilitating state transitions during facial affect processing. Finally, we uncover spatial relationships (i) between regional variation in differential control energy (alprazolam *versus* placebo) and (ii) both serotonin and dopamine neurotransmitter systems, indicating that alprazolam may exert its effects by altering neuromodulatory systems. Together, these findings provide a new perspective on the distributed emotion processing network and the effect of GABAergic modulation on this network, in addition to identifying an association between schizophrenia risk and abnormal GABAergic effects on persistence energy during threat processing.

## Introduction

Schizophrenia is associated with deficits in emotion processing. Individuals with schizophrenia demonstrate marked deficits in facial affect perception, as measured through tasks that require the identification of emotions such as happiness, sadness, anger or fear [1, 2]. Emotion processing deficits in schizophrenia contribute substantially to impairments in social cognition and poor functional outcomes [3, 4]. First-degree family members of individuals with schizophrenia also display abnormalities in facial affect perception, albeit to a lesser extent than probands [5–8]. Abnormalities in first-degree relatives are particularly remarkable as the study of family members allows for the investigation of schizophrenia associated endophenotypes without the confounding effects of antipsychotic medication and secondary effects related to disease chronicity [9]. More broadly, investigations of facial affect processing in family members may offer insight into a key cognitive domain adversely affected by schizophrenia and can serve to inform effective treatment strategies.

Prior studies have used neuroimaging to characterize the neural circuitry associated with altered facial affect processing in individuals with schizophrenia and their relatives. These studies have primarily focused on linking differences in activation of limbic regions like the amygdala with altered identification and recall of threat-related faces [9–12]. However, facial affect perception is a complex process involving multiple brain regions, and evidence exists for impairment in both emotion-processing limbic regions as well as early-stage visual processing in schizophrenia [9, 13]. Facial affect processing involves a distributed network comprising limbic regions, fusiform and occipital cortex, medial and lateral prefrontal areas, and insula [14–16]. Indeed, components of this distributed network have been implicated in facial emotion processing abnormalities in individuals with schizophrenia [17] and individuals with high genetic risk for schizophrenia [18], suggesting heritability. Thus, an integrative understanding of facial affect processing abnormalities in schizophrenia requires analysis of the distributed network regulating a complex domain.

Facial affect processing abnormalities in schizophrenia, and other cognitive deficits, may be driven by abnormal GABAergic neurotransmission [19]. Notably, GABAergic abnormalities in schizophrenia have been documented quite broadly, across the prefrontal cortex [20], visual cortex [21, 22], amygdala [23], and temporal lobe [24], regions that overlap with the distributed network involved in facial affect processing. The role of GABAergic circuitry in facial affect processing and its impairment in schizophrenia can be effectively studied through pharmacological modulation using GABA modulators like benzodiazepines [25]. Alprazolam (Xanax®) is among the most widely used benzodiazepines, with well-known anxiolytic effects through enhanced GABAergic inhibition of the amygdala and limbic structures, and sedative effects from more broad GABAergic inhibition [26–28]. Thus, benzodiazepine challenge provides an opportunity to study the role of GABAergic circuitry in the etiology of facial affect processing abnormalities in schizophrenia.

Benzodiazepines impair emotion identification and emotion memory in healthy individuals, mainly in processing threatening faces [29–31]. The neural basis for the observed impairments in threat processing have been investigated in neuroimaging studies of emotion processing with benzodiazepine challenge in healthy subjects. These studies have shown that benzodiazepines alter activation of brain regions in the distributed facial affect processing network including amygdala, fusiform gyrus, orbitofrontal cortex, and insula during facial affect processing tasks [32, 33]. We showed that alprazolam unmasks amygdalar and/or GABAergic abnormalities in first-degree relatives of individuals with schizophrenia during emotion identification and recall tasks [34]. However, there remains a lack of mechanistic understanding of benzodiazepine action during facial affect processing that goes beyond traditional activation studies. More recent tools for modeling the dynamics of brain activation states can help to synthesize results from activation studies and provide mechanistic insight into benzodiazepine action as well as GABAergic abnormalities in schizophrenia.

The mechanistic basis of benzodiazepine action on the brain during emotion processing can be effectively modeled using network control theory (NCT). NCT is a tool originating in theoretical physics and systems engineering that has successfully been used to understand how to control real-world systems comprised of interacting components, such as power grids and electronic circuits [35, 36]. In the context of NCT, control refers to the ability to drive the system, through a suitable choice of inputs, from an initial state to a final state. Given that the brain is a complex system comprised of interconnected networks of neurons [37], NCT provides an intuitive and compelling tool to model the dynamic trajectory of brain activation states that support its rich cognitive functions. Indeed, NCT has already been used to provide insight into the structure and function of model nervous systems like *C. elegans* [38], *Drosophila* [39], mouse [39, 40], and macaque [41], as well as human brain networks [39, 42–46].

The application of NCT to model the brain typically involves the definition of a structural network through diffusion weighted imaging, and the definition of brain states as activation patterns across brain regions [47]. Brain states can be defined by arbitrarily switching ‘on’ canonical brain sub-networks like the visual and default mode networks, or directly as task activation obtained through functional magnetic resonance imaging data [43, 48–51]. The NCT framework is then used to model the temporal progression of brain states as a function of the underlying structural network and theoretical control energy applied to different brain regions. The calculated control energy may represent external electrical stimulation or internal cognitive control needed to steer the brain between defined initial and final states [42]. Additionally, the brain regions important for driving specific brain state transitions can be identified through control impact analysis. This framework naturally lends itself to modeling the effect of drugs like alprazolam in driving brain state trajectories relevant to facial affect processing and can provide mechanistic insight into the mode of action of the drug beyond simple measures of activation.

Here we applied principles from network control theory to investigate the effect of alprazolam and schizophrenia risk status in driving brain state transitions during facial affect processing. We leveraged a previously reported dataset [34] where fMRI BOLD data was collected during emotion identification and emotion memory tasks, with and without administration of alprazolam, in a cohort consisting of healthy controls and unaffected first-degree relatives of individuals with schizophrenia. We considered task-evoked brain activation patterns during emotion processing tasks to be brain states and quantified the theoretical control energy needed to maintain those states – the persistence energy. In our previous study, we showed that alprazolam reduced amygdala activation during emotion identification only in first-degree relatives, suggesting an unmasking of amygdala GABAergic hypersensitivity in this group [34]. Accordingly, our primary hypothesis was that when administered alprazolam, family members would have altered persistence energy during identification and recall of threatening faces which requires amygdalar processing, but not during non-threatening or neutral stimuli. We predicted that brain regions of high control impact in the NCT model would align with known regions involved in facial affect processing including fusiform cortex, occipital cortex, and sub-cortical regions like the amygdala and insula. Finally, we predicted that regions of high control impact would also spatially align with regions of high GABA receptor expression, but not with other neurotransmitters like dopamine and serotonin, reflecting the biological mode of benzodiazepene action. By testing and validating our hypotheses, we uncover novel insights regarding the network organization of emotion processing and the contribution of GABAergic abnormalities toward emotion processing deficits associated with genetic risk for schizophrenia.

## Methods

### Participants

The sample included 27 healthy participants with a first-degree relative affected by schizophrenia and 20 healthy controls without a family history of schizophrenia, for a total of *n* = 47 participants. Controls and relatives were matched based on demographic and clinical variables (Table 1). After excluding scans based on motion estimates (mean framewise displacement > 0.5 mm), the final sample for data analysis included *n* = 44 participants (19 relatives; 25 controls) for emotion identification, and *n* = 40 participants (17 relatives; 23 controls) for emotion memory (see Supplementary Methods for details on assessment). Study procedures were approved by the University of Pennsylvania Institutional Review Board, and written informed consent was obtained from participants. Participants underwent standard medical, neurological, psychiatric, and neurocognitive evaluations (see Supplementary Methods).

**Table 1:** Demographic and clinical information at time of scan.

Variable	Controls (*n* = 27)		Relatives (*n* = 20)		*p*-value	
	Percentage	Proportion	Percentage	Proportion		Odds ratio
Sex (% F)	51.9	14 F/13 M	55.0	11 F/9 M	1.0	0.88
Handedness (% R)	92.6	25 R/2 L	80.0	16 R/4 L	0.38	0.32
Smoke (% N)	77.8	21 N/6Y	80.0	16 N/4Y	1.0	0.88
	Mean (SD)	Range	Mean (SD)	Range		Test statistic* (DOF)
Age (years)	39.0 (11.4)	21.1–56.5	42.3 (14.8)	20.9–59.4	0.31	−1.02
Education (years)	15.0 (2.0)	11.0–19.0	14.8 (2.3)	12.0–20.0	0.79	0.26 (45)
Parental education	13.6 (3.1)	7.5–20.0	13.9 (2.7)	9.5–18.0	0.76	−0.31 (43)
Height (in.)	67.7 (4.0)	61.0–77.0	67.6 (4.3)	60.0–73.0	0.93	0.09 (45)
Weight (lb.)	176.4 (33.0)	115.0–255.0	175.5 (34.0)	118.0–250.0	0.93	0.09 (45)
BMI (lb./in.^2^)	27.1 (4.9)	20.4–36.8	27.0 (4.5)	18.7–33.9	0.94	0.08 (45)
Trait anxiety	28.3 (6.7)	20.0–47.5	30.1 (8.9)	20.0–58.0	0.64	−0.46
Schizotypy (SIS) total	11.5 (7.2)	1.0–29.0	15.0 (7.2)	7.0–39.0	0.11	−1.64 (44)
Alprazolam level (ng/mL)	7.5 (4.1)	0.0–13.0	7.8 (4.1)	1.0–14.0	0.80	−0.26 (42)

*F* Female, *M* Male, *R* Right, *L* Left, *N* Non-smoker, *Y* Smoker, *SD* Standard deviation, *BMI* Body mass index, *SIS* Structured interview for schizotypy, *DOF* Degrees of freedom; reported *p*-values are from Fisher’s exact test for categorical variables (sex, handedness, and smoking status), Wilcoxon rank sum tests for non-normal data (age, trait anxiety), and two-sample t-tests for normally distributed data (all other variables). *t-statistic for two-sample t-tests, z-statistic for Wilcoxon rank sum test (degrees of freedom not reported).

### Study design and pharmacological challenge

To study the impact of GABAergic modulation on brain activation during emotion processing, participants underwent fMRI imaging during facial affect processing tasks with and without administration of alprazolam. Details of study design have been described previously [34]. Briefly, participants underwent two identical fMRI sessions approximately one week apart. Participants were administered 1 mg oral alprazolam in one session and an identical-appearing placebo in the other session, in a balanced double-blind within-subject crossover design.

**Fig. 1:**
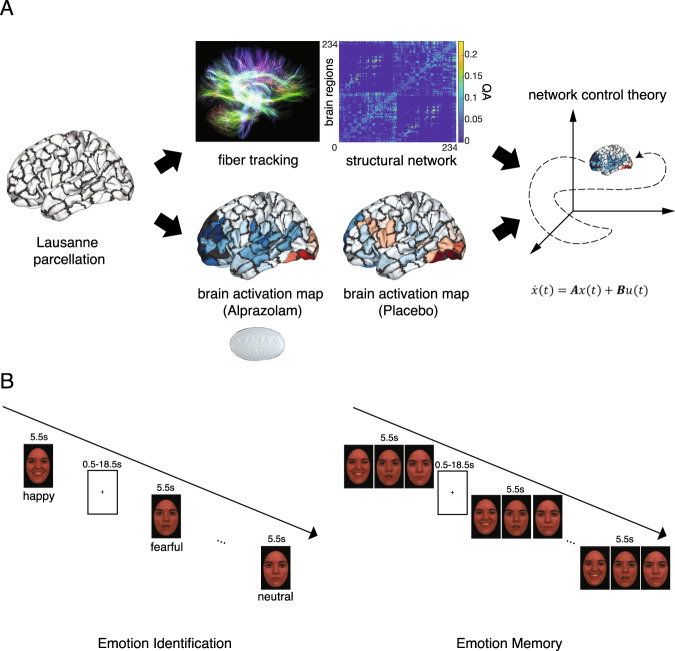
Operationalizing network control theory in the context of human neuroimaging. **A** The strength of structural connections between brain regions were determined by the quantitative anisotropy (QA) estimated from diffusion spectrum imaging data. We used beta coefficients from general linear models to specify brain activation maps during task sessions where participants were given 1 mg oral alprazolam or placebo. These maps were then fed into a network control model to analyze the energy required for transitions between different brain states. We were particularly interested to estimate the persistence energy, ***P***_***e***_, defined as the energy required to maintain a state. Brain regions in the cortex and subcortex were defined by the 234-node Lausanne parcellation. **B** Schematic of emotion identification and emotion memory tasks. In the emotion identification task, participants were required to identify the emotions expressed on the displayed faces, with variable crosshair fixation between cues. In the emotion memory task, the same sequence of faces as in the preceding emotion identification task was presented, with each target face accompanied by two foil expressions. Participants were instructed to recall the expression that matched the previously seen face.

During each fMRI session, participants performed an emotion identification task followed by an emotion memory task (Fig. 1B). In the emotion identification task, 60 unique color pictures of human faces were presented in pseudorandomized order, with facial expressions falling into one of five emotional categories: happy, sad, fearful, angry or neutral [52]. Participants were asked to identify the emotion expressed on each face. In the emotion memory task, the same sequence of faces as in the preceding emotion identification task was presented, with each target face accompanied by two foil expressions. Participants were instructed to recall the expression that matched the previously seen face. In both tasks, each emotion category was presented 12 times, with each emotion being used as a foil 24 times in the emotion memory task. Faces were displayed for 5.5 s, with a variable interval of between 0.5 and 18.5 s, during which a complex crosshair matched to faces on perceptual qualities was presented. Each task lasted 10.5 min, with a 2 min delay between tasks.

### Image acquisition and processing

Structural and functional image sequences were acquired with a Siemens Trio 3 T system (Erlangen, Germany). Structural images were acquired for the whole brain, whereas functional volumes were acquired in a slab covering ventral regions of the brain with a spatial resolution of 2 × 2 × mm (Fig. S1).

We used *fMRIPrep* software (version 1.2.6) to process the BOLD fMRI data [53]. Briefly, *fMRIPrep* was used to perform brain extraction and segmentation of individual T1-weighted images, spatial normalization of T1 images to the ICBM 152 Nonlinear Asymmetrical template, susceptibility distortion correction for BOLD images, estimation of confound variables including head motion parameters and resampling of BOLD sequences to MNI152NLin2009cAsym standard space. We excluded sessions for which the average framewise displacement was greater than 0.5 mm. No other exclusion criteria were applied.

Next, we used generalized linear models (GLM) to measure subject-specific brain activation patterns during emotion identification and memory tasks. Specifically, GLM analysis was performed using the FEAT module [54] in FSL 5.0.10 implemented using XCP Engine [55]. BOLD sequences preprocessed using *fMRIPrep* were high-pass filtered (100 s) and spatially smoothed (4 mm FWHM, isotropic); further, the first 6 non-steady state volumes were discarded. All event conditions were modeled as 5.5s-boxcars convolved with a canonical hemodynamic response function. Consistent with previous work [15, 56], correct responses to fear and anger stimuli were combined as a “threat” regressor; happy and sad stimuli were combined as a “non-threat” regressor; and neutral stimuli were modeled separately. All specified contrasts measured BOLD activation compared to baseline. Incorrect responses and 6 motion parameters were included as regressors of non-interest. We chose to include only correct responses in the model to limit the potential effects of inattention due to sedation by alprazolam.

We then divided the brain into 233 parcels based on the Lausanne parcellation (after excluding the brain stem), which provides coverage of both cortical and subcortical areas including thalamus, caudate, putamen, pallidum, accumbens, hippocampus, and amygdala [57]. Parameter estimates (beta weights) from each voxel were averaged within each parcel resulting in estimates of brain activation (brain states); these activation maps were then evaluated using network control theory.

See Supplementary Methods for further details on image acquisition and processing, as well as a detailed explanation of the network control theory model used.

### Construction of structural brain networks from diffusion spectrum imaging data

Structural brain connections are an essential component of network control theory models. Since we did not collect structural brain images in our previous study, we leveraged an average structural matrix from a separate study. Diffusion spectrum imaging (DSI) data was collected from a separate set of 10 healthy young adults as described elsewhere [47]. Consistent with previous work [48, 49], we defined nodes of the structural network as brain regions according to the Lausanne atlas [57]. To encode each structural network, we constructed adjacency matrices for each subject based on the quantitative anisotropy (QA) between each pair of brain regions. The average structural matrix across 10 participants was used for all results shown in the main text.

See Supplementary Methods for further details on DSI image acquisition, processing, and structural matrix generation.

### Spatial correlations with neurotransmitter maps

In order to explore the underlying biology of drug action reflected through control energy measures, we analyzed the spatial alignment of drug-induced differences in control energy input with neurotransmitter receptor maps obtained through PET imaging. Given the known role of alprazolam as a GABA modulator [27], we expected brain regions whose control energy input varied strongly with drug condition to also be correlated with GABA receptor density, but not with other receptors such as serotonin and dopamine.

For this analysis, we used published PET/SPECT maps of the following receptors: 5-HT1a (serotonin 5-hydroxytryptamine receptor subtype 1a), 5-HT1b (5-HT subtype 1b), 5-HT2a (5-HT subtype 2a), D1 (dopamine D1), D2 (dopamine D2), DAT (dopamine transporter), F-DOPA (dopamine synthesis capacity), GABA_A_ (gamma-aminobutyric acid A receptor), NAT (noradrenaline transporter), and SERT (serotonin transporter) [58–64]. All provided PET/SPECT maps were voxel-wise average group maps of variable numbers of healthy volunteers, linearly rescaled to a range of 0 to 100 [58]. We further averaged the PET/SPECT maps voxel-wise for each Lausanne parcel to obtain 233×1vectors, each of which represented a spatial map of the distribution of a given neurotransmitter. We then evaluated correlations between all PET/SPECT maps and region-wise difference maps in control input between alprazolam and placebo sessions for all subjects.

See Supplementary Methods for details on statistical analysis.

## Results

### Alprazolam differentially modulates persistence energy in relatives and controls during threat emotion processing

**Fig. 2:**
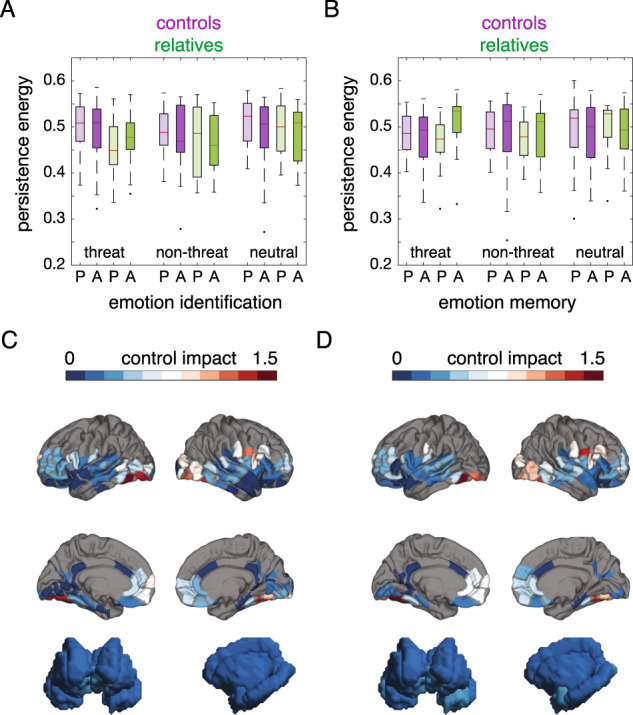
Alprazolam modulates persistence energy during recall of threatening faces. **A** Boxplots show persistence energy for the emotion identification task, grouped by emotion category; P Placebo; A Alprazolam. **B** Boxplots show persistence energy for emotion memory task, grouped by emotion category. We observed a significant group**×**drug interaction in the threat condition (***γ***_**11**_ = −0.048, *p* = 0.026, df = 73); P Placebo, A Alprazolam. **C** Average spatial maps of control impact for threat emotion identification, shown on surface renderings of cortical and subcortical areas. **D** Average spatial maps of control impact for threat emotion identification, shown on surface renderings of cortical and subcortical areas. Parcels outside the imaging slab are colored gray.

We first tested our primary hypothesis of alprazolam altering persistence energy during threat emotion processing. Persistence energy was measured as the control energy needed to maintain specific brain activation patterns observed during in-scanner emotion identification and memory tasks. We evaluated the effect of group and drug on persistence energy using linear mixed models with drug and group treated as categorical variables (Equations 1−3, Supplementary Methods). During emotion identification, there was no main effect of group or drug, and no group×drug interaction in any emotion category (Fig. 2A, see Supplementary Data Files 1 for model coefficients and statistics). During emotion memory, we found that persistence energy was moderately increased with alprazolam administration during recall of threat stimuli, in family members but not in controls (Fig. 2B, group×drug interaction, *γ*_11_ = −0.048, *p* = 0.026, df = 73). There was no main effect of group (*γ*_01_ = 0.026, *p* = 0.133, df = 73) or drug (*β*_1*i*_ = 0.01, *p* = 0.50, df = 74) (Supplementary Data Files 1). As expected, no significant effects were found in non-threat and neutral conditions. Alternate analyses where categorical drug indicator was replaced with alprazolam blood levels and categorical group indicator was replaced with the total score on the structured interview for schizotypy (SIS) showed similar results (Supplementary Information, Supplementary Data Files 2, 3).

In order to elucidate the influence of structural brain networks and spatial activation patterns on the observed results, we performed a series of investigations using structural and spatial null models (see Supplementary Methods for details). When control energies were recalculated using null models of structural brain networks, we found that the new mixed model coefficients remained largely similar to the coefficients from the original model (Fig. S4, Supplementary Data Files 8). However, when control energies were recalculated using null models of spatial activation patterns, we found large deviations in the mixed model coefficients compared to our original model (Fig. S5, Supplementary Data Files 9). These investigations showed that the differential effect of alprazolam on persistence energy in relatives and controls during recall of threatening faces was partially dependent on the underlying structural brain networks but largely dependent on the task-specific spatial activation patterns.

Finally, we used control impact analysis to investigate the relative importance of different brain regions in driving brain state transitions associated with emotion identification and memory. Control impact of individual nodes was measured by iteratively removing each node from the network and recomputing the persistence energy [49].

As hypothesized, we found that regions with high control impact in both emotion identification and memory tasks were primarily located in the fusiform and occipital cortex, reflecting the visual and facial processing nature of the tasks (Fig. 2C, D, Supplementary Data Files 4). One node in the lateral precentral gyrus also exhibited high control impact, perhaps reflecting facial motor control processes involved in embodied aspects of facial affect perception [65, 66]. Surprisingly, subcortical areas including the amygdala, hippocampus, and insula had relatively low control impact. Areas with high control impact aligned largely with areas of high activation obtained from beta weight maps estimated from a general linear model (Fig. S3, Supplementary Data Files 5). Similar high associations were found between region-wise control energy integrated over simulation time and GLM beta weights. Thus, the control model suggests that the direct and indirect connectivity of the fusiform and occipital regions with the whole brain structural network facilitates efficient coordination of neural dynamics associated with facial affect processing.

### Individual differences in persistence energy explain variance in task performance during threat emotion identification

Next, we performed an exploratory analysis to examine the relationship between persistence energy and task performance. We reasoned that increased persistence energy during emotion identification and memory tasks might reflect cognitive effort expended and thus might be reflected in measures of task performance. We summarized task performance using an efficiency measure (accuracy divided by reaction time), and then evaluated associations between efficiency and persistence energy using a different set of linear mixed effects models (Equations 4–6, Supplementary Methods).

**Fig. 3:**
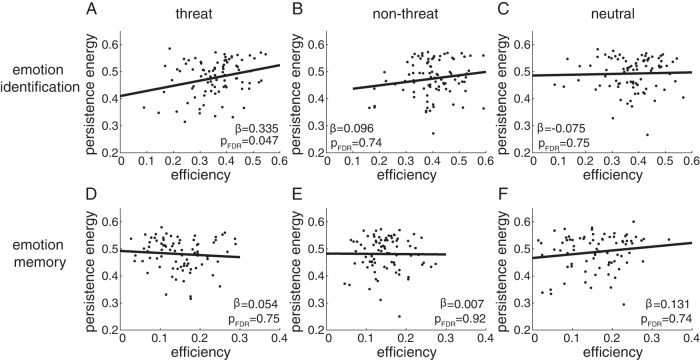
Task performance during threat emotion identification can be predicted from persistence energy. Scatterplots of efficiency in task performance against persistence energy, shown for the emotion identification (**A**–**C**) and emotion memory (**D**–**F**) tasks, separately for the threat, non-threat, and neutral categories. Task performance efficiency is measured as the proportion of correct responses divided by the median reaction time for correct responses. The ***β*** weights from the linear mixed effects models containing drug, group, age, and sex as covariates are shown on each plot, along with associated *p*-values corrected for multiple comparisons. Associations are significant for threat emotion identification at a significance level of p_FDR_ < 0.05.

We found that efficiency during threat emotion identification was positively associated with persistence energy (Fig. 3A, main effect of persistence energy, β = 0.335, p_FDR_ = 0.047, df = 81). No significant associations were found for other emotion categories, or for the emotion memory task, after correction for multiple comparisons (Fig. 3B–F). Consistent with our previous study [34], we found that alprazolam significantly reduced task efficiency during both emotion identification and memory tasks (see Supplementary Data Files 6 for all model coefficients and statistics). We also found, consistent with our previous study, that there were no group effects on task performance for any of the task conditions (Supplementary Data Files 6). Thus, relatives and controls performed equally well on emotion identification and memory tasks.

### Regional differences in control energy spatially align with neurotransmitter systems

**Fig. 4:**
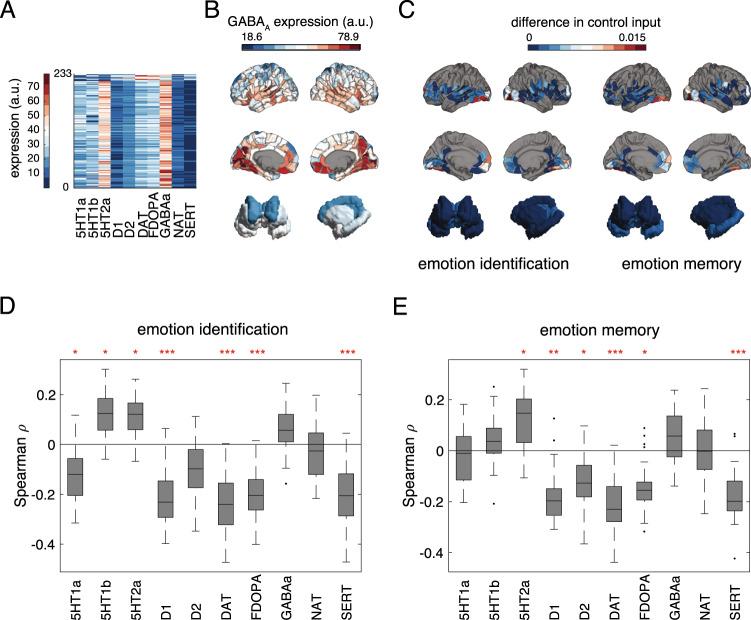
Neurotransmitter receptor profiles are associated with drug effect on control input. **A** PET neurotransmitter heatmaps from Dukart et al. (2021). **B** PET map of GABA_A_ expression shown on surface renderings of cortical and subcortical areas. **C** Regional differences in average control input on alprazolam and placebo (absolute values) during threat emotion identification and memory, shown on surface renderings of cortical and subcortical areas. **D**, **E** Boxplots of subject-level Spearman correlation coefficients between PET spatial maps and regional control input differences during threat emotion ID (**D**) and threat emotion memory (**E**). Red asterisks indicate the level of statistical significance from permutation tests with 500 permutations, corrected for multiple comparisons; *p_FDR_ < 0.05, **p_FDR_ < 0.005, ***p_FDR_ < 0.0005.

Finally, we explored the underlying biology of control energy measures by evaluating the spatial correspondence between control energy parameters from our model and known neurotransmitter systems described through PET/SPECT receptor maps [58]. To achieve this, we calculated the spatial correlation between PET/SPECT maps (Fig. 4A, B) and maps describing regional differences in control energy input between alprazolam and placebo conditions (Fig. 4C, Supplementary Data Files 7). Given the known role of alprazolam as a GABA_A_ receptor modulator [27], we expected brain regions whose control input varied strongly with drug condition to also be correlated with GABA_A_ receptor density.

To calculate regional control input difference maps, we subtracted total control input in each brain region (over the simulation time) between alprazolam and placebo sessions. These maps show that the effects of alprazolam are mainly located in occipital and fusiform areas, with some effects in frontal and orbitofrontal regions (Fig. 4C, Supplementary Data Files 7). We then evaluated correlations between regional control input difference maps and PET/SPECT receptor maps. Surprisingly, we found that correlations between control input difference maps and GABA_A_ receptors were not significant (Fig. 4D, E). Moreover, in both emotion identification and memory tasks, control input difference maps were positively correlated with serotonergic receptors, and negatively correlated with dopaminergic receptors (Fig. 4D, E).

## Discussion

In this study, we applied a network control theory model to investigate the effects of alprazolam during facial affect processing in a cohort of healthy controls and first-degree relatives of people with schizophrenia. The main findings from our analysis and their implications are discussed below.

### Control energy measures reflect GABAergic abnormalities in schizophrenia

In our previous study [34], we found that alprazolam effects on standard task fMRI measures in amygdala were stronger in relatives of individuals with schizophrenia compared to controls during emotion identification, suggesting alprazolam could be unmasking underlying GABAergic abnormalities. Given these prior results, we expected that alprazolam would also differentially influence control energy measures associated with whole-brain emotion-processing activation patterns in relatives versus controls. Indeed, we found that alprazolam increased the persistence energy associated with brain states during the recall of threatening faces (anger and fear) in family members but not in controls.

The persistence energy is the control energy needed to maintain a brain state associated with a task and has been previously associated with the cognitive effort required during those tasks [43]. We found further evidence for this relation between energy and effort by demonstrating that increased persistence energy is associated with better task performance in a subset of tasks. Since family members demonstrated relatively normal behavioral performance, increased persistence energy during the recall of threatening faces may represent a compensatory GABAergic mechanism that enables them to perform as well as controls. Further investigation into potential compensatory mechanisms might uncover promising avenues to target therapeutic drugs that aim to support improved cognitive function in schizophrenia.

In our previous study [34], we found that alprazolam induced strong reduction in amygdala activation during emotion identification (but not during emotion recall) in relatives compared to controls. In the current manuscript, we report increased persistence energy during threat emotion recall (but not during emotion identification) in relatives compared to controls. While the findings from our previous study shed light on amygdalar GABAergic abnormalities in relatives, the findings from the current study provide insight into GABAergic abnormalities in the broad facial affect processing network. It is possible that the more purely affective nature of the emotion identification task renders it more sensitive to changes in amygdalar activation [11], which explains the main finding from our previous study. Persistence energy is a measure that reflects not only regional activation but also the energetic stability of a particular pattern of regional activation given the underlying structural network. The emotion recall task being cognitively demanding [11] may be more sensitive to brain-wide measures such as persistence energy, which explains the main finding of the current study.

Control energy measures such as persistence energy go beyond traditional measures of activation, instead reflecting brain-wide network dynamics constrained by underlying white matter architecture [43, 50, 51]. Since facial affect processing is known to involve a distributed network of brain regions [14, 15], models that capture network-wide brain dynamics are important to investigate the neural substrate of this cognitive domain and its modulation by psychiatric disease. Our results add evidence of GABAergic abnormalities in family members when processing faces with negative affect, unmasked by drug action. Importantly, these abnormalities were measured using network-wide readouts, demonstrating that our analyses provide an important complementary approach to identifying such effects. Taken together, our results indicate that control energy measures reflect network-wide effects of GABAergic abnormalities during facial affect processing in first-degree relatives of individuals with schizophrenia.

### Regions most impacting network control align with the facial affect processing network and distributions of neuromodulatory receptors

We sought to understand the impact of different brain regions in driving brain state transitions associated with emotion processing, expecting that regions of high importance would align with the distributed network associated with facial affect processing [14–16]. In partial support of this hypothesis, we found that brain regions with high control impact during emotion identification and memory were primarily in the fusiform and occipital cortices. Fusiform and visual brain regions are core components of the classical network associated with facial affect processing [14, 15, 67, 68]. Our mathematical model suggests that the direct and indirect connectivity of these regions with the whole brain structural network facilitates efficient coordination of neural dynamics associated with performance of face processing behavior, providing novel intuition regarding their role as the “face areas” of the brain.

Our analysis also showed that limbic and sub-cortical regions such as amygdala, hippocampus and insula did not have high control impact in any emotion category. These regions have been classically associated with emotion processing [16, 69], and their low prominence in our network control model is therefore somewhat surprising. Our results suggest that primary sensory areas associated with visual processing exert top-down control on whole-brain activation during facial affect processing, while subcortical regions including the amygdala are circumscribed to a bottom-up role with limited impact on the rest of the brain. It is possible that changes in amygdala activation, while being significantly different between relatives and controls during emotion identification [34], did not produce a significant change in persistence energy due to the limited role of this brain region in our model. Further, the high prominence of fusiform and occipital regions and low prominence of subcortical regions is also consistent with a constructive view of emotion [70] – the perception of faces constructs a multi-modal explanation of the sensory stimuli and context, triggering an emotion reflected in the instance of emotion depicted in the face stimuli.

Finally, we sought to understand the underlying biology of alprazolam action during facial affect processing by evaluating correlations between drug-induced differences in control input and neurotransmitter receptor maps obtained through PET/SPECT imaging. Due to alprazolam’s known mechanism of action as a positive allosteric modulator of GABA_A_ receptors [27], we expected drug-induced differences in control input to align spatially with GABA_A_ receptors. We found that these correlations, although trending positive, were not statistically significant. However, drug difference maps were positively correlated with serotonergic receptors and negatively correlated with dopamine receptors. These results indicate that the effect of alprazolam may manifest primarily through driving complementary serotonergic and dopaminergic neuromodulatory systems [71–73], perhaps shedding light on a possible mechanistic basis of its well-documented sedative and anxiolytic effects. Our results align with previous studies which have shown that benzodiazepines like most drugs do not act in isolation, and their clinical effects likely result from affecting multiple interacting neurotransmitter systems [73]. Overall, our findings and approach highlight the utility of network control theory in understanding the neurobiological basis of drug action in the brain.

### Limitations

This study has a number of limitations. The first relates to the sample under study. As discussed previously [34], the sample studied did not include patients with schizophrenia, preventing definitive evaluation of putative endophenotypes. Further, this particular sample of relatives did not exhibit marked emotion processing abnormalities assessed by behavioral performance (Supplementary Data Files 6), unlike previous results with larger cohorts [8]. Schizotypy scores of first-degree relatives in our sample were not statistically different from healthy controls (*p* = 0.11). However, this could also be due to the low sample size (Cohen’s d effect size ~0.48). Further, we did not find significant associations between schizotypy scores and control energy measures, perhaps reflecting the lack of significant variation in clinical risk for psychosis in this sample. The small sample size also resulted in uncertainty in our finding of altered persistence energy during threat emotion memory, given the relatively low statistical significance of this result [74]. Thus, future work could seek to replicate the findings reported here in larger samples, and control energy abnormalities found here in family members could be tested in patients with frank illness. Second, while GLM parameter estimates provide a reliable indicator of brain activation patterns at the group level in response to task stimuli, this approach fails to account for dynamic variations in activation, including latencies in interactions among different brain regions. Recently developed network approaches could prove useful in studying the effect of drug and schizophrenia status on these dynamics [75, 76]. Third, the use of a group-averaged structural matrix and PET/SPECT maps from external datasets is a limitation of our study. Individuals with schizophrenia and their relatives are known to have altered structural brain properties and receptor distributions [20, 77–79]. It is possible that group differences in structural brain properties would have contributed to more significant group differences in control energy in our model. However, as demonstrated by our null model analysis, structural brain networks appear to have minimal influence over control energy measures, leading us to believe that any further contributions to group differences would be minimal. Regarding group differences in receptor distributions, it remains to be seen whether the resolution of PET/SPECT imaging modalities is sufficiently high to capture group differences in receptor distributions between healthy controls and first-degree relatives. Fourth, while the alprazolam dose in this study (1 mg) is sufficient to produce clinical and neurobiological effects, the lack of dose response in the experimental design weakens the causal and mechanistic interpretation of the findings [80], and should be assessed in future studies. Finally, we were able to analyze neuroimaging data only from a limited slab that was chosen for high-resolution coverage of a specific set of emotion processing areas including fusiform and orbitofrontal cortex in addition to subcortical and limbic regions. The power of the network control approach in uncovering whole-brain network dynamics was thus limited to regions covered within the slab. Future studies could extend the network control approach to whole-brain imaging data obtained during facial affect processing.

## Conclusion

In summary, we used a novel network control theory framework to investigate abnormalities in the distributed facial affect processing network in individuals at familial risk for schizophrenia. Using this modeling framework, we found that alprazolam increased persistence energy in relatives but not in controls during recall of threatening faces. Increased persistence energy during threat recall points toward altered dynamics within the facial affect processing network in relatives during threat processing elicited by alprazolam, suggesting a compensatory mechanism during processing of threatening faces. Our approach and results provide a new perspective and deeper theoretical understanding of the neural mechanisms underlying facial affect processing, and point toward specific altered dynamics during facial affect processing in individuals at genetic risk for schizophrenia.

More broadly, the network control approach described here is a powerful mechanistic framework to uncover endophenotypes of psychiatric disease and to investigate the effect of pharmacologic manipulation on the brain. Brain regions identified by the network control approach can be used to inform more targeted drug development for neuropsychiatric disorders, in addition to informing novel regions for stimulation through paradigms such as rTMS [81]. Further, control energy measures represent a readout of brain function and can be used to investigate abnormalities in various cognitive domains such as working memory [43] and sensorimotor function [82].

### Citation diversity statement

Recent work in several fields of science has identified a bias in citation practices such that papers from women and other minority scholars are under-cited relative to the number of such papers in the field [83–87]. Here we sought to proactively consider choosing references that reflect the diversity of the field in thought, form of contribution, gender, race, ethnicity, and other factors. First, we obtained the predicted gender of the first and last author of each reference by using databases that store the probability of a first name being carried by a woman [87, 88]. By this measure (and excluding self-citations to the first and last authors of our current paper), our references contain 7.23% woman(first)/woman(last), 12.66% man/woman, 22.09% woman/man, and 58.01% man/man. This method is limited in that a) names, pronouns, and social media profiles used to construct the databases may not, in every case, be indicative of gender identity and b) it cannot account for intersex, non-binary, or transgender people. Second, we obtained predicted racial/ethnic category of the first and last author of each reference by databases that store the probability of a first and last name being carried by an author of color [89, 90]. By this measure (and excluding self-citations), our references contain 12.63% author of color (first)/author of color(last), 13.13% white author/author of color, 19.37% author of color/white author, and 54.87% white author/white author. This method is limited in that a) names and Florida Voter Data to make the predictions may not be indicative of racial/ethnic identity, and b) it cannot account for Indigenous and mixed-race authors, or those who may face differential biases due to the ambiguous racialization or ethnicization of their names. We look forward to future work that could help us to better understand how to support equitable practices in science.

### Supplementary information

Supplementary Information

Supplementary Data Files 1-9

## Data Availability

All analysis code is available at https://github.com/arunsm/alpraz-project.git.
